# Ibudilast for alcohol use disorder: study protocol for a phase II randomized clinical trial

**DOI:** 10.1186/s13063-020-04670-y

**Published:** 2020-09-11

**Authors:** Elizabeth M. Burnette, Wave-Ananda Baskerville, Erica N. Grodin, Lara A. Ray

**Affiliations:** 1 Department of Psychology, University of California Los Angeles, Los Angeles, CA USA; 2 Interdepartmental Program for Neuroscience, University of California Los Angeles, Los Angeles, CA USA; 3 Department of Psychiatry and Biobehavioral Sciences, University of California Los Angeles, Los Angeles, CA USA

**Keywords:** Alcohol use disorder, Ibudilast, AUD, PDE4, fMRI, Stress, Cue-reactivity

## Abstract

**Background:**

Alcohol use disorder (AUD) is a chronic and relapsing condition for which current pharmacological treatments are only modestly effective. The development of efficacious medications for AUD remains a high research priority with recent emphasis on identifying novel molecular targets for AUD treatment and to efficiently screen new compounds aimed at those targets. Ibudilast, a phosphodiesterase inhibitor, has been advanced as a novel addiction pharmacotherapy that targets neurotrophin signaling and neuroimmune function.

**Methods:**

This study will conduct a 12-week, double-blind, placebo controlled randomized clinical trial of ibudilast (50 mg BID) for AUD treatment. We will randomize 132 treatment-seeking men and women with current AUD. We will collect a number of alcohol consumption outcomes. Primary among these is percent heavy drinking days (PHDD); secondary drinking outcomes include drinks per day, drinks per drinking day, percent days abstinent, percent subjects with no heavy drinking days, and percent subjects abstinent, as well as measures of alcohol craving and negative mood. Additionally, participants will have the option to opt-in to a neuroimaging session in which we examine the effects of ibudilast on neural activation to psychosocial stress and alcohol cues. Finally, we will also collect plasma levels of proinflammatory markers, as well as subjective and biological (salivary cortisol) markers of stress response.

**Discussion:**

This study will further develop ibudilast, a safe and promising novel compound with strong preclinical and clinical safety data for AUD, and will probe biological mechanisms underlying the effects of Ibudilast on stress, neuroinflammation, and alcohol cue-reactivity and craving. If ibudilast proves superior to placebo in this study, it will set the stage for a confirmatory multi-site trial leading to FDA approval of a novel AUD treatment.

**Trial registration:**

ClinicalTrials.gov NCT03594435 “Ibudilast for the Treatment of Alcohol Use Disorder”. Registered on 20 July 2018.

## Administrative information

The order of the items has been modified to group similar items (see http://www.equator-network.org/reporting-guidelines/spirit-2013-statement-defining-standard-protocol-items-for-clinical-trials/).

**Table:** 

Title {1}	**Ibudilast for Alcohol Use Disorder: Study protocol for a phase II randomized clinical trial**
Trial registration {2a and 2b}.	**ClinicalTrials.gov, NCT03594435 “Ibudilast for the Treatment of Alcohol Use Disorder”, registered July 20, 2018. See Supplementary Table **1**for all items in the WHO trial registration data set for this study.**
Protocol version {3}	**Protocol Version 8, created September 2019, IRB-approved October 8, 2019.**
Funding {4}	**NIH NIAAA R01AA026190 – “A RANDOMIZED CONTROLLED CLINICAL TRIAL OF THE NEUROIMMUNE MODULATOR IBUDILAST FOR THE TREATMENT OF ALCOHOL USE DISORDER”**
Author details {5a}	**Department of Psychology, University of California, Los Angeles, Los Angeles, CA (EMB, WAB, ENG, LAR)** **Brain Research Institute, University of California, Los Angeles, Los Angeles, CA (EMB, LAR)** **Department of Psychiatry and Biobehavioral Sciences, University of California, Los Angeles, Los Angeles, CA (ENG, LAR)**
Name and contact information for the trial sponsor {5b}	**Trial Sponsor: Lara A. Ray, PhD. University of California, Los Angeles, Psychology Department, 1285 Franz Hall, Box 951563, Los Angeles, CA 90095-1563, USA. E-mail address:** **lararay@psych.ucla.edu**
Role of sponsor {5c}	**Medicinova provides the study medication. The funder and Medicinova had no role in the design, analysis, interpretation, or writing of the report.**

## Introduction

### Background and rationale {6a}

Alcohol use disorder (AUD) is a chronic and relapsing condition for which current pharmacological treatments are only modestly effective [1]. The development of efficacious medications for AUD remains a high research priority with recent emphasis on identifying novel molecular targets for AUD treatment and to efficiently screen new compounds aimed at those targets [2, 3]. To that end, modulation of neuroimmune function represents a promising novel target for AUD [4]. Chronic alcohol consumption produces a sustained inflammatory state, such that individuals with AUD have increased neuroinflammation throughout the brain [5], and alcohol-induced neuroinflammation is thought to contribute to chronic alcohol seeking behavior and to the behavioral and neurotoxic effects of alcohol [6]. In rodents, lipopolysaccharide-induced neuroinflammation produces prolonged increases in alcohol consumption [7], and knocking out neuroimmune signaling genes attenuates alcohol preference and self-administration [8]. Therefore, a medication that reduces proinflammatory signaling may produce anti-alcohol and neuroprotective effects that may be beneficial for the treatment of AUD.

Ibudilast (IBUD; aka, MN-166, previously AV411) has been advanced as a novel addiction pharmacotherapy that targets neurotrophin signaling and neuroimmune function. IBUD inhibits phosphodiesterases 4 (PDE4) and 10 (PDE10) and macrophage migration inhibitory factor (MMIF) [9]. As PDE4 and MMIF are critically involved in proinflammatory signaling [10, 11], and PDE10 negatively regulates neurotrophin expression [12], the inhibition of these molecules by IBUD has been theorized to reduce neuroinflammation and promote neurotrophin expression [9]. In support, IBUD enhances neurotrophin expression, reduces pro-inflammatory cytokine release, and attenuates neuronal death [13]. In rodents, IBUD has been demonstrated to reduce ethanol intake by approximately 50% both under conditions of maintenance and relapse testing [14]. These recent preclinical findings for IBUD support prior studies indicating pharmacological inhibition of PDE4 and PDE10 decreases alcohol intake [15–17].

To advance medications development for AUD, our laboratory has recently completed a randomized, double-blind, placebo-controlled crossover laboratory study of IBUD in non-treatment seeking individuals with AUD (NCT02025998) [18]. This study tested the safety, tolerability, and initial human laboratory efficacy of IBUD (50 mg BID) on measures of subjective response to alcohol, as well as cue- and stress-induced changes in craving and mood. Twenty-four individuals completed two separate 7-day intensive outpatient protocols which included daily visits for medication administration and testing. Upon reaching a stable target dose of IBUD (or matched placebo), participants completed a stress-exposure session, an alcohol cue-exposure session, and an IV alcohol administration session. Results indicated that IBUD was well tolerated and associated with mood improvements during stress- and alcohol-cue exposures, and with reduction in tonic levels of alcohol craving. Exploratory analyses revealed that among individuals with higher depressive symptomatology, IBUD attenuated the stimulant and positive mood-altering effects of alcohol.

Given the promising pre-clinical and initial human laboratory findings, we will conduct a large-scale randomized clinical trial (RCT) of IBUD (50 mg BID) in treatment-seeking participants with AUD. Additionally, we will collect psychosocial stress- and alcohol-cue-related neuroimaging data as part of the trial. As an exploratory aim, we will also collect proinflammatory biomarkers from participants over the course of the trial.

## Objectives {7}

### Primary aims

#### Primary aim 1

To test whether IBUD (50 mg BID) will decrease percent heavy drinking days (PHDD; HDD defined as 5+ drinks for men and 4+ for women), as compared to placebo, over the course of the 12-week trial.

#### Primary aim 2

To test the efficacy of IBUD (50 mg BID) on secondary alcohol consumption endpoints, namely (a) drinks per day, (b) drinks per drinking day, (c) percent days abstinent, (d) percent subjects with no heavy drinking days, and (e) percent subjects abstinent, as well as measures of alcohol craving and negative mood, over the course of the 12-week trial.

#### Primary aim 3

To determine the effect of IBUD on neural activation to alcohol cues and psychosocial stress. Participants will complete a neuroimaging paradigm in which they view alcoholic and non-alcoholic beverage cues and will rate their subjective craving for alcohol. They will also complete a psychosocial stress neuroimaging task and rate their subjective stress. The primary outcome variables will be blood-oxygen-level-dependent (BOLD) activation to alcohol cues and psychosocial stress.

### Secondary aims

#### Exploratory aim 1

To test whether the effects of IBUD (50 mg BID) on the primary and secondary endpoints (aims 1 and 2) are moderated by depressive symptomatology. This is based on our finding that IBUD attenuated the stimulant effects of alcohol among individuals with higher levels of depressive symptomatology.

#### Exploratory aim 2

To test whether IBUD (50 mg BID), compared to placebo, reduces neuroinflammation, as indexed by circulating blood levels of proinflammatory markers over the course of the 12-week trial.

#### Exploratory aim 3

To evaluate the relationship between neural alcohol cue-reactivity and drinking outcomes, by creating linear mixed models to assess the relationship between percent heavy drinking days and ventral striatum neural alcohol cue-reactivity.

#### Exploratory aim 4

To evaluate the effect of IBUD on changes in stress after exposure to a stress-inducing fMRI paradigm, by collecting subjective measures of stress and salivary cortisol in order to measures biological stress responses.

## Trial design {8}

The study design consists of a 12-week, double-blind, placebo-controlled randomized clinical trial of IBUD (50 mg BID) for the treatment of AUD. We will randomize 132 treatment-seekers with current AUD over the course of 4 years and will collect neuroimaging data from 64 of them. As a behavioral support platform, all participants will complete the NIAAA-developed and computer-delivered program “Take Control” during the study, an intervention consisting of 11 computerized modules that provide evidence-based, field tested information for individuals with alcohol problems, and suggestions for making changes in their drinking. Participants will complete telephone screening, followed by in-person eligibility assessment, a physical exam for medical eligibility, randomization to study medication or matched placebo, and in-person follow-up visits at 4, 8, and 12 weeks of treatment. A brain imaging session will take place at week 4 (if participant is eligible). In addition, timeline follow-back (TLFB) assessment of drinking outcomes will occur by telephone on weeks 2, 6, and 10. A final safety check visit will occur on week 16, consisting of repeated clinical labs and ECG.

## Methods: participants, interventions and outcomes

### Study setting {9}

All aspects of the study will take place in the city of Los Angeles, California, in the USA. All screening and testing research participants will be conducted in the Primary Investigator’s Addictions Research Laboratory, in the Psychology Department at the University of California, Los Angeles (UCLA). The clinical labs and ECGs for the entire research protocol will be conducted at the outpatient unit of the Westwood/UCLA Clinical Translational Research Center (CTRC). In addition, the brain imaging session will be performed at the Center for Cognitive Neuroscience located on the UCLA campus.

### Eligibility criteria {10}

Inclusion criteria for participants are as follows: (1) age between 18 and 65; (2) meet current (i.e., past 12 months) DSM-5 diagnostic criteria for alcohol use disorder moderate or severe; (3) treatment-seeking for AUD; and (4) report drinking at least 28 drinks per week if male (21 drinks per week if female) in the 28 days prior to consent. In order to bolster the heavy drinking status of the sample, at least 50% of the sample will be comprised of very heavy drinkers (defined as ≥ 35 drinks/week for men and ≥ 28 drinks/week for women).

Exclusion criteria for participants are as follows: (1) a current (last 12 months) DSM-5 diagnosis of substance use disorder for any psychoactive substances other than alcohol and nicotine; (2) a lifetime DSM-5 diagnosis of schizophrenia, bipolar disorder, or any psychotic disorder; (3) positive urine screen for narcotics, amphetamines, or sedative hypnotics; (4) clinically significant alcohol withdrawal symptoms as indicated by a score ≥ 10 on the Clinical Institute Withdrawal Assessment for Alcohol-Revised (CIWA-Ar); (5) pregnancy, nursing, or refusal to use reliable method of birth control (if female); (6) a medical condition that may interfere with safe study participation (e.g., unstable cardiac, renal, or liver disease, uncontrolled hypertension or diabetes); (7) AST, ALT, or GGT ≥ 3 times upper normal limit; (8) attempted suicide in the past 3 years and/or serious suicidal intention or plan in the past year; (9) currently on prescription medication that contraindicates use of IBUD, including alpha or beta agonists, theophylline, or other sympathomimetic; (10) currently on any medications for AUD or any psychotropic medications (e.g., psychostimulants and benzodiazepines) with the exception of stable antidepressants (stable dose for ≥ 4 weeks); or (10) other circumstances that, in the opinion of the investigators, compromises participant safety.

Exclusion criteria for the fMRI component of the trial are as follows: (1) non-removable ferromagnetic object in body; (2) claustrophobia; and (3) serious head injury or prolonged period of unconsciousness (> 30 min). Participants are able to opt out of the brain imaging session at their own discretion.

During the initial screening visit, the DSM-5 diagnostic criteria for a current (past 12-month) AUD diagnosis (moderate or severe) as well as exclusionary diagnoses (i.e., lifetime psychosis) is assessed using the structured clinical interview for DSM-5 (SCID-5) and will be performed by a master’s level clinician under the supervision of the PI. If participants appear to be eligible after the initial screening visit, they will be scheduled for the second screening visit with the study physician. The study physician will review each participant’s medical history, vital signs, weight, review of systems, and laboratory tests including liver function tests (LFTs), drug screen, chemistry screen, and urine pregnancy screen to determine if it is medically safe for the participants to take study medication.

### Who will take informed consent? {26a}

At the initial in-person screening visit and prior to conducting any research-related procedures, a trained member of the study team will conduct the three-part consent process which details the procedures that take place during the screening process. First, participants will be asked to read and provide verbal consent for the breathalyzer. If the breathalyzer test is 0.000 g/dl, study staff will read and discuss the written informed consent outlining procedures for the initial screening visit with the participant. Once the participant has asked questions and has a clear understanding of the procedures, the participant will sign the consent form. If the participant is found eligible to continue to the medical screening visit, a second written consent form outlining the study purpose, procedures, potential risks, and benefits will be reviewed and signed in the presence of the study physician in a private, confidential setting. Only physician investigators who are continuously involved in the research and qualified to answer questions regarding the nature of the subject’s participation and the alternatives to participation will obtain the second informed consent. Participants will be provided a copy of both the first and second informed consent.

### Additional consent provisions for collection and use of participant data and biological specimens {26b}

At the first initial in-person screening visit, participants will read and sign a consent form that outlines the procedures for collecting biological specimens such as a urine sample for a toxicology screen and pregnancy test (if applicable) at each study visit. At the medical screening visit, participants will sign the experimental consent form that includes the electrocardiogram (ECG) and blood sample for a comprehensive metabolic panel (CMP) and complete blood cell count (CBC) in order to evaluate overall health and determine eligibility. In addition, participants will be asked to consent to the collection of biological specimens during the study such as a blood sample for neuroinflammation assays at every study visit, salivary cortisol samples, and brain imaging at the week 4 visit. Participant data and biological specimens used to evaluate eligibility and study compliance (i.e., urine sample, ECG, CBC, CMP) will be disposed of after testing. Data and specimens germane to the research study such as de-identified neuroinflammation assays and salivary cortisol samples will be owned by the University of California or by a third party designated by the University (such as another university or a private company). Participants will be asked to indicate if they permit part of the sample to be shared with other researchers and/or used in future studies. However, the samples for this study will be used for the specified analyses and will not be stored for secondary analysis.

## Interventions

### Explanation for the choice of comparators {6b}

The trial is placebo-controlled, due to there being no universal standard-of-care medication for AUD treatment. Since the standard treatment for AUD is therapist-delivered behavioral support, all subjects will be provided the computer-based, NIAAA-developed Take Control program. This decision is supported by a recent study [19] comparing computer-delivered Take Control to Therapist-Delivered Platforms (TDP), which found comparable drinking outcomes and higher medication adherence in the Take Control trials, suggesting that Take Control is a comparable and cost-efficient alternative to TDP in AUD clinical trials.

### Intervention description {11a}

Medication and matched placebo will be supplied by MediciNova. The study drug is IBUD (MN-166, previously known as AV411), and the formulation is 10-mg delayed-release Pinatos® capsules, the Japanese generic IBUD product produced by Taisho Pharmaceuticals and imported by MediciNova. The target dose will be 50 mg BID (5 × 10 mg capsules twice daily). To minimize nausea, IBUD’s most common side effect, all participants will begin at 20 mg BID for 2 days increasing to 50 mg BID on day 3 and remaining at the 50 mg BID dosing until week 12. For the last 3 days of week 12, participants will reduce the dose (step down procedure) to 20 mg BID prior to stopping the medication at the end of the study. The medication will be in blister packaging. Each blister pack will have 2 weeks’ worth of medication. At the end of the randomization visit, participants will receive three blister packs of medication, approximately 6 weeks of medication (half of the study medication). Participants will receive the second half of their medication during the week 4 in-person visit.

### Criteria for discontinuing or modifying allocated interventions {11b}

The study physicians will be available to participants for the entire duration of the study. The study physicians will call every participant at the end of the first week on the study medication to discuss and manage any adverse events. Study staff will also notify the study physicians of any adverse events recorded during the follow-up visits (4, 8, and 12 weeks post randomization). Side effects will be collected through an open-ended question asking participants to report any adverse events they may be experiencing. If study medication adjustments (i.e., drug dose change) are required due to safety concerns, it is up to the study physicians’ discretion to make a dose adjustment or terminate medication. Medication will be stopped based on pre-specified criteria for discontinuation of study medication: (a) development of agitation, hostility, depressed mood, or changes in behavior or thinking not typical of alcohol use or withdrawal (more severe and/or temporally un-related to alcohol use/withdrawal; (b) severe nausea and vomiting; (c) have a systolic blood pressure greater than 160, or a diastolic blood pressure greater than 100 (i.e., cutoffs for stage 2 hypertension), or a heart rate greater than 70% of the maximum heart rate expected for their age [0.70(220 − age)]; (d) females who become pregnant; (e) > 50% increase in AST/ALT at any of the LFT assessments (weeks 4, 8, and 12); and (f) any circumstances that, in the opinion of the investigators, compromise participant safety. Four weeks after medication is terminated (week 16) a follow-up safety visit will be conducted and will consist of clinical labs and ECG, which will be reviewed by the study physician.

### Strategies to improve adherence to interventions {11c}

To assist with adherence to intervention protocols, participants receive a detailed medication log that lists the date, study day blister pack number, and AM/PM tablet number for everyday of the study in order for the participant to keep track of medication consumption. In addition, participants are provided a document with tips on how to remember to take medication twice a day. Compliance will be monitored by the study staff using the pill count method at each follow-up visit. Finally, participants will work with the same research staff member throughout the trial and will complete regular check-ins with that staff member.

### Relevant concomitant care permitted or prohibited during the trial {11d}

Participants are deemed ineligible if they are currently prescribed medication that contraindicates the use of IBUD, including alpha or beta agonists, theophylline, or other sympathomimetic. Participants are also deemed ineligible if they are on any medication for AUD or any psychotropic medication (e.g., psychostimulants and benzodiazepines) with the exception of stable antidepressants (stable dose of ≥ 4 weeks). All medication taken by the participant 2 weeks prior to the initial in-person screening visit are collected and reviewed by the study physician at the medical screening visit. Study staff will continue to record new medication on a source document via participant self-report through the final follow-up at week 12 and will notify the study physician of any new medication for physician review. Concomitant participation in AA meetings and other recovery activities are not encouraged, but not prohibited either.

### Provisions for post-trial care {30}

A follow-up safety visit will be conducted 4 weeks after medication is terminated (week 16). Clinical labs and ECG will be repeated, which will be reviewed and managed by the study physician as a final safety check. After study termination (week 16), unresolved adverse events will be followed up by the study physician for a minimum of 14 days unless the investigator’s judgment dictates otherwise, the event has resolved or stabilized prior to the 14-day period, or the subject is lost to follow-up. If the unresolved AE is an ongoing pregnancy, it must be followed to conclusion.

### Outcomes {12}

#### Primary aims

##### Primary aim 1

Percent heavy drinking days (PHDD; HDD defined as 5+ drinks for men and 4+ for women) will be computed using timeline follow-back (TLFB) data over the course of the trial.

##### Primary aim 2

Secondary alcohol consumption endpoints include the following: (1) drinks per day, (2) drinks per drinking day, (3) percent days abstinent, (4) percent subjects with no heavy drinking days (PSNHDD), and (5) percent subjects abstinent, all computed from the TLFB data over the course of the trial.

##### Primary aim 3

The main contrasts of interest for this outcome will be BOLD activation differences during alcohol vs. non-alcoholic beverage blocks of the cue-reactivity task, and BOLD activation differences during stress vs. control blocks of the psychosocial stress task.

#### Secondary aims

##### Exploratory aim 1

Secondary outcomes of alcohol craving and depressed mood will be captured by the PACS and BDI-II, respectively. Physical dependence (presence of tolerance/withdrawal) will also be examined.

##### Exploratory aim 2

Serum samples will include innate immune receptors (TLR2, TLR4), cytokines (TNF-α, IL-1α, IL-1β, IL-2, IL-6, IL-10, IL12p70, GM-CSF, IFNγ), chemokines (MCP-1, MIP-1α, and MIP-1β), and other inflammatory signaling molecules (reactive oxidative species, NO, substance P, C-reactive protein).

##### Exploratory aim 3

The primary measures for this outcome will be percent signal change BOLD activation in the alcohol vs. non-alcoholic beverage blocks of the alcohol cue-reactivity task within an anatomically defined region of interest in the ventral striatum, and PHDD as measured in primary aim 1.

##### Exploratory aim 4

Subjective (Subjective Distress Units Scale (SUDS) and Spielberger State-Trait Anxiety Inventory (STAI)) and biological (salivary cortisol) measures of stress response will be collected prior to and after the neuroimaging stress paradigm.

### Participant timeline {13}

**Fig. 1:**
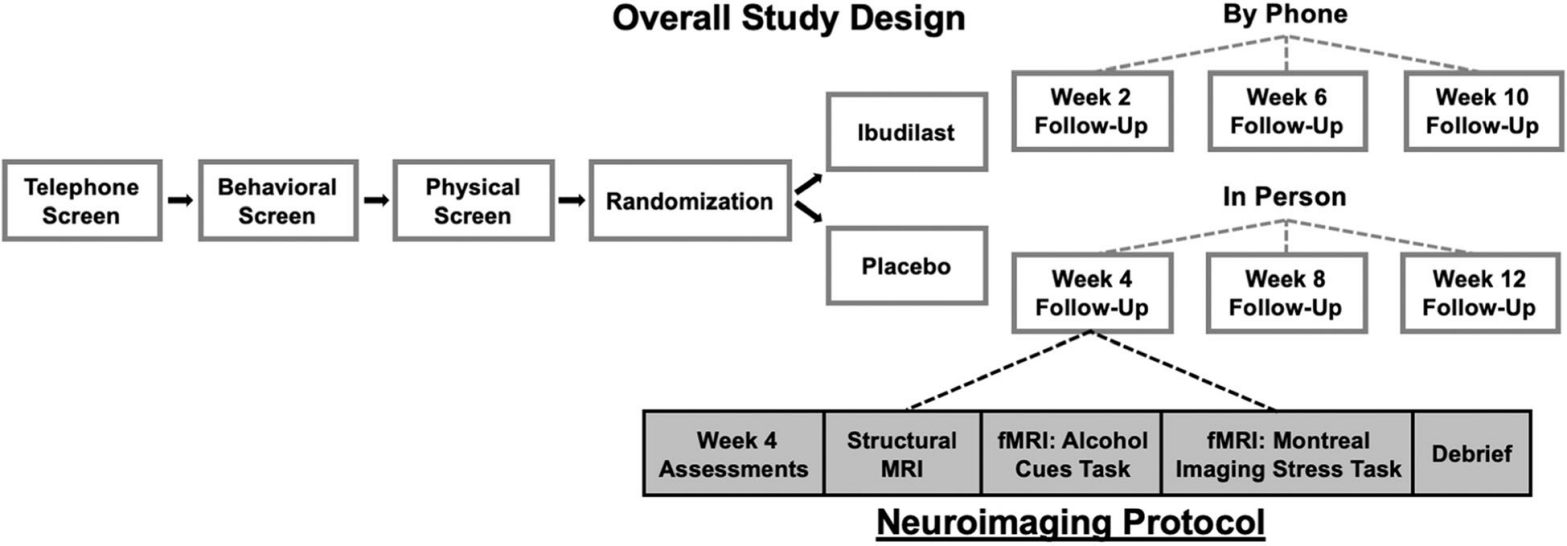
Schematic diagram of overall study design

Participants will initially be screened over the phone. Eligible individuals will complete in-person screening at which we will administer the Structured Clinical Diagnostic Interview for DSM-5 (SCID-5 [20]), individual differences measures, and collect a urine sample for toxicology and pregnancy for females. Eligible participants will complete a physical exam and laboratory tests. Participants will then be randomized to an intervention (i.e., IBUD or placebo). We will continue to follow up with the participants over the course of the 12-week study as follows: over the phone in weeks 2, 4, and 6, and in-person for weeks 4, 8, and 12. The overall study design is outlined in Fig. 1. Assessments administered at each visit are detailed in Table 1. At the week 4 follow-up visit, participants who choose to undergo the neuroimaging protocol will complete, in addition to the main study assessments, the neuroimaging protocol, including a structural MRI, alcohol cue-reactivity task, and a psychosocial stress task.

**Table 1:** Procedures and measures administered at each study visit

Study visit: Procedure	Initial Screen	Med Screen	Rand. Visit	Wk 4 F/U	Wk 8 F/U	Wk 12 F/U	Wk 16 F/U
Informed Consent: Initial Screen	x						
Informed Consent: Experimental/Medical		x					
Adverse Childhood Experience Questionnaire (ACE)	x						
Adverse Events/Serious Adverse Events (AE/SAE)		x	x	x	x	x	
Alcohol Breathalyzer	x	x	x	x	x	x	
Alcohol Dependency Scale (ADS)	x			x	x	x	
Alcohol Purchase Task (APT)	x			x	x	x	
Alcohol Use Disorders Identification Test (AUDIT)	x						
Beck Anxiety Inventory (BAI)	x		x	x	x	x	
Beck Depression Inventory-Revised (BDI-II)	x		x	x	x	x	
Birth Control Assessment	x						
Brain Imaging (if eligible)				x			
Brief Trauma Questionnaire (BTQ)	x						
Clinical Institute W/D Assessment for Alcohol (CIWA-AR)	x		x	x	x	x	
Columbia Suicide Severity Rating Scale (C-SSRS)	x		x	x	x	x	
Safety Labs CMP/CBC (blood sample)		x					x
Concomitant Medications	x	x	x	x	x	x	
Cannabis Use Disorder Identification Test (CUDIT)	x						
Demographics	x						
Drinking Goal	x						
Drug Compliance/Accountability				x	x	x	
Drug Screen (urine sample)	x	x	x	x	x	x	
Electrocardiogram (ECG)		x					x
Fagerstrom Test for Nicotine Dependence (FTND)	x						
Family Tree Questionnaire (FTQ)	x						
Graded Chronic Pain Scale (GCPS)	x			x	x	x	
ImBIBe	x		x	x	x	x	
Inflammation and Behavior Questionnaire			x	x	x	x	
Insomnia Severity Index (ISI)	x		x	x	x	x	
Locator Form	x						
Medical History		x					
NIH Toolbox			x			x	
Neuroimmune assays (blood sample)			x	x	x	x	
Penn Alcohol Craving Scale (PACS)	x		x	x	x	x	
Physical Exam		x					
Pregnancy Test (urine sample)	x		x	x	x	x	
Profile of Mood States (POMS)	x		x	x	x	x	
Readiness to Change (RTC) Ladder	x						
Reward-Relief Drinking Scale	x						
Risky Families Questionnaire	x						
Salivary Cortisol Testing				x			
Spielberger State-Trait Anxiety Scale (STAI)				x			
Structured Clinical Interview for DSM-5 (SCID-5)	x						
Subjective Distress Units Scale (SUDS)				x			
Take Control (Computer-delivered)			x	x	x	x	
Timeline Follow Back (TLFB)	x	x	x	x	x	x	x
Vital Signs (including weight)	x	x	x	x	x	x	x

### Sample size {14}

A power analyses was performed using the PASS 14 software for the primary hypothesis under the mixed effect model design. Because the lack of preliminary data on IBUD for the primary outcome, we used a similarly designed clinical trial of varenicline [21] as a reference for the anticipated effect size in our power calculations. Specifically, the varenicline study [21] showed that the percent heavy drinking days was 39.6 (SE = 3.7) for the varenicline versus 50.2 (SE = 3.6) for placebo (difference = 10.6; Cohen’s *d* = 0.31). With a total of 132 participants (66 subject/group * 2 groups), our repeated measures mixed effect model design will have 93.73% power to detect an effect size of 0.3 (between a small effect size of 0.2 and medium effect size of 0.5 (126)) for the treatment group difference with 12 repeated measurements assuming a compound symmetry covariance structure when the standard deviation is 35.0, the correlation between observations on the same subject is 0.2, and the alpha level is 0.05. We also investigated the power under a variety of other scenarios by varying the covariance structure (Compound Symmetry, Simple, AR(1), and Banded(1)) and the within-subject correlation (from 0.1 to 0.4), all yielded satisfactory power (ranging from 79 to 99%). Importantly, power analyses have been performed to ensure that the study has sufficient power to detect a small to medium effect size for testing the primary hypothesis using a mixed effects model that takes into account of the repeated measures design.

The neuroimaging analyses will evaluate if IBUD alters neural alcohol cue or stress reactivity in 64 treatment-seeking individuals with AUD (*n* = 32/medication group). The projected power is adequate for this project as recent between-subject designs using neuroimaging and pharmacotherapies for AUD [22–25] have enrolled 20–30 participants per cell. Preliminary analysis of the alcohol cues task suggests adequate signal detection in mesocorticolimbic circuitry and reliable signal from this task has been detected in as few as 10 participants [26]. Of note, power will be lower for the drinking outcomes analysis as participants will likely display a range of drinking patterns. As such, the drinking outcomes aim has been listed as exploratory.

### Recruitment {15}

Participants will be recruited from the community radio, newspaper advertisements, Craigslist advertisements, posting flyers around west Los Angeles, and continual posting on Clinical Connections. Campaigns in local busses and metro trains will also be implemented. Through text and visuals, recruitment materials will invite individuals who wish to change their drinking and who indicate they have a drinking problem. Confidence in recruitment strategies achieving adequate participant enrolment is based on past success in recruitment efforts at our site.

## Assignment of interventions: allocation

### Sequence generation {16a}

Randomization will be done in a 1:1 ratio, to either IBUD or placebo using a stratified block randomization procedure gender and heavy drinking (moderate drinking defined as ≥ 14 drinks/week for men and ≥ 27 drinks/week for women versus very heavy drinking defined as ≥ 28 drinks/week for men and ≥ 21 drinks/week for women) as the stratification factors. The allocation sequence is computer-generated and performed by the study statistician, who is not involved in participant enrollment.

### Concealment mechanism {16b}

A blinded stratification list, including subject ID, scenario number, gender, drinking status, and sequence is used to assign participants a scenario number and sequence number based on the stratification criteria (gender and drinking status). The pharmacist matches this information to the unblinded stratification list and fills the prescription. Only the pharmacist has access to the unblinded stratification list. Both the experimental medication and matched placebo are prepared as white capsules and blister packaged to look the same. The experimental medication and matched placebo are taken by mouth for a total of 12 weeks. Participants will take two capsules of either the experimental medication or matched placebo twice a day for the first 2 days. Starting on day 3, they will take 5 capsules twice a day until week 12. For the last 3 days of week 12, participants will take 2 capsules twice a day prior to stopping the medication at the end of week 12.

### Implementation {16c}

Prior to the start of the study, an allocation sequence is generated. From this sequence, the pharmacist labels the scenario of ibudilast and placebo as an A or B. Only the pharmacist knows if the active medication or placebo is labeled A or B. This list is then blinded by the laboratory manager into a blank document and includes a scenario and sequence number. After the medication screening visit, the study physician will determine medical eligibility for the study. If eligible, participants will be randomized in a 1:1 ration, to either IBUD or placebo using a stratified block randomization procedure, gender and drinking status as stratification factors. Based on the blinded stratification list, participants will be assigned to a sequence and scenario number. The sequence and scenario number will be listed on the prescription that is sent to the pharmacist. The pharmacist will use the sequence and scenario number along with the unblinded stratification list to fill the prescription with either the active medication or the placebo. Eligible participants will be scheduled to pick up their prescription. Once the participant has received their prescription, the study coordinator will enroll the participant to the trial using OnCore, a clinical trial management system (CTMS) used at UCLA.

## Assignment of interventions: blinding

### Who will be blinded {17a}

The experimental design is a double-blind clinical trial. Both study participants and research staff will not know if the participant is receiving the placebo or active medication. The placebo is prepared as a white capsule and is blister packaged to look the same as the active medication. In order to eliminate any distinction from the active medication, the placebo is taken by mouth twice a day. All data analysis will be performed blinded to medication allocation.

### Procedure for unblinding if needed {17b}

In the event that significant medical problems are encountered, the blind will be broken and appropriate medical treatment will be provided. Significant medical problems are defined as any of the following severe adverse events defined per the US FDA as any fatal event, any immediately life-threatening event, any permanent or substantially disabling event, any event that requires or prolongs inpatient hospitalization, or any congenital anomaly, or any unexpected adverse drug experiences that have not previously been observed. In addition, any other important medical event that a study investigator judges to be severe because it may jeopardize the subject’s health in some manner or require intervention to prevent one of the above outcomes, or which would suggest a significant hazard, contraindication, side effect, or precaution. The PI will promptly report all severe adverse events or unexpected adverse drug experiences as required by the UCLA Institutional Review Board (IRB), the NIAAA Program Officer, the study’s DSMB, and the FDA (where appropriate). A decision to break the blind will be determined by the severity of the medical problem and its relevance to study medication.

## Data collection and management

### Plans for assessment and collection of outcomes {18a}

A battery of measures designed to provide a comprehensive assessment of medical safety, alcohol and cigarette use, other drug use, and psychological measures are included (Table 1). In order to substantially decrease the task of managing the data stream, most self-reported study measures will be collected using Qualtrics. The following interviews and self-report measures will be administered during the initial (in-person) screening visit for the purposes of assessing eligibility and measuring relevant individual differences: (1) The 30-day timeline follow-back (TLFB) interview [27] measures quantity and frequency of drinking; (2) the Structured Clinical Interview for DSM-5 (SCID [20]) will be performed by a master’s level clinician using under the supervision of the PI. The SCID-5 will be used to assess current (past 12-month) AUD diagnosis (moderate or severe) as well as exclusionary diagnoses (e.g., lifetime psychosis); (3) Clinical Institute Withdrawal Assessment for Alcohol (CIWA-AR) is a brief 10-item measure that assesses for the emergence of alcohol withdrawal symptoms. The CIWA-AR has been used both in clinical and research applications and has demonstrated both reliability and validity [28]; (4) the Columbia Suicide Severity Rating Scale (C-SSRS) [29], interview assess suicide ideation, intensity of ideation, and suicidal behavior. C-SSRS is intended for use by trained administrators who have completed the required online training certification and who will be supervised by the PI; (5) smoking is assessed by (a) number of cigarettes per day/any form of tobacco and (b) the Fagerstrom Test for Nicotine Dependence (FTND) [30]; (6) Cannabis Use Disorder Identification Test (CUDIT) to identify persons with hazardous and harmful patterns of cannabis consumption [31]; (7) the Graded Chronic Pain Scale (GCPS), a 7-item measure used to evaluate an individual’s overall severity of chronic pain from pain that has lasted at least 6 months; (8) the Profile of Mood States (POMS) for measuring dimensions of mood [32]; the Beck Anxiety Inventory (BAI) [33] a self-report assessment to measure anxiety and depression levels used widely in clinical trial; (9) The Beck Depression Inventory, Revised (BDI-II) [34] captures depressive symptomatology (and is needed to test exploratory aim 1); (10) the Adverse Childhood Experience Questionnaire (ACE), a 10-item self-report measure developed to identify childhood experiences of abuse and neglect [35]; (11) information on family history of alcohol problems will be collected using the Family Tree Questionnaire (FTQ); (12) the Penn Alcohol Craving Scale (PACS) [36] will provide an assessment of tonic levels of craving for alcohol; (13) the Alcohol Dependence Scale (ADS), a scale measuring alcohol dependence symptoms over the past 12 months [37]; (14) the Alcohol Purchase Task (APT), a 16-item scale that uses hypothetical situations regarding alcohol purchases and consumptions at varying prices in order to generate several indices of alcohol-related reinforcement; (15) the Alcohol Use Disorders Identification Test (AUDIT) to identify persons with hazardous and harmful patterns of alcohol consumption [38]; (16) Readiness to Change (RTC) ladder is a measure with 11 responses items to assess motivation to reduce or cut back on drinking [39]; (17) the Reward-Relief Drinking Scale [40] is a 4-item scale that measures an individual’s reward drinking tendencies; (18) the Insomnia Severity Index (ISI) [41] measures sleep quality; and (19) the ImBIBe is a 15-item questionnaire in which the subject responds on a 5-point scale responses to questions on the consequences of alcohol use (this is an adapted version from the DrInc-2R) [42]. Breath alcohol concentration (BrAC), urine drug screen, and pregnancy test (for females) will be administered at each study visit. A BrAC = 0.00 g/dl will be required for participation in each study visit.

Medical eligibility will be determined by the study physicians using the following assessments: (a) review of participant’s medical history; (b) laboratory tests (i.e., LFTs, gamma-glutamyl transferase (GGT), glucose, drug screen, chemistry screen, and pregnancy test); (c) physical examination, including vital signs, weight, and review of systems; and (d) electrocardiogram (ECG). For safety reasons, clinical lab tests will be repeated at weeks 4, 8, and 12. And the ECG will be repeated at week 12. Lastly, participants will return 1 month post medication discontinuation (week 16) to repeat all clinical labs and ECG as a final safety check.

Several individual differences measures obtained during the initial screening visit will be repeated during randomization visit as well as the monthly assessments to allow for analyses of change overtime and as a function of study medication (IBUD vs. Placebo). The TLFB interview will be conducted by telephone on weeks 2, 6, and 10 to complement the in-person assessments on weeks 4, 8, and 12 and to shorten the duration between drinking outcomes assessment. This will improve data quality and maintain contact with research participants on a more frequent basis in order to prevent dropout.

In addition to the measures testing the study outcomes, measures of neuroinflammation and brain imaging will be collected. Serum samples will be collected at randomization and at 4, 8, and 12-week follow-ups to address exploratory aim 2. Due to the diurnal rhythm of cytokine production, samples will be collected at the same time of day for all subjects (12:00 PM–1:00 PM). Assayed markers will include innate immune receptors (TLR2, TLR4), cytokines (TNF-α, IL-1α, IL-1β, IL-2, IL-6, IL-10, IL12p70, GM-CSF, IFNγ), chemokines (MCP-1, MIP-1α, and MIP-1β), and other inflammatory signaling molecules (reactive oxidative species, NO, substance P, C-reactive protein).

The neuroimaging session, completed during the week 4 follow-up visit, includes 2 neuroimaging paradigms: (1) the alcohol cues task and (2) the Montreal Imaging Stress Task (MIST). The neuroimaging paradigms will be presented in counterbalanced order between participants.

Neuroimaging will be conducted using a 3-T Siemens Prisma Fit MRI scanner at the UCLA Center for Cognitive Neuroscience. Scanning parameters for functional magnetic resonance imaging (fMRI) scanning will be as follows: TR, 2 s; TE, 30 ms; flip angle, 90°; FOV, 192 mm; matrix, 64 × 64; voxel size, 3 × 3 × 4mm^3^; slice thickness, 4 mm; and 34 slices. A T2-weighted, high resolution, matched bandwidth, anatomical scan (MBW), and a magnetization-prepared rapid-acquisition gradient echo (MPRAGE) will be acquired to enable registration (TR, 1.9 s; TE, 2.26 ms; FOV, 250 mm; matrix, 256 × 256; sagittal plane; slice thickness, 1 mm; 176 slices). The orientation for MBW and functional scans will be oblique axial to maximize brain coverage. During data acquisition, head restraints will be placed using a foam pillow.

The alcohol cues task is well-validated, with strong reliability and within-participant stability [26]. There are four types of cues: alcoholic beverages, non-alcoholic beverages, blurred images, and a fixation cross. Stimuli are presented in six 120-s epochs (total scan duration, 12 min), with each epoch consisting of four 24-s blocks (one block of alcohol cues, one block of non-alcohol beverage cues, one block of blurred images, and one block of fixation). During each 24-s block, 5 individual pictures will be displayed for ~ 4.8 s each. Alcohol blocks will be specific to beverage type (beer, wine, or liquor), with 2 blocks of each beverage type. Each block will be followed by a 6-s washout period. Subjects will provide ratings of their alcohol craving following each cue block.

The MIST is a block design fMRI paradigm in which participants solve mental arithmetic problems [43]. There are three conditions: stress induction, control, and rest, which are presented pseudo-randomly. During the control and stress induction conditions, participants are asked to solve mental arithmetic problems of varying degrees of difficulty and are given feedback on their performance (correct or incorrect). During the stress induction condition, 2 performance indicators are displayed on a colored bar to induce social evaluative threat: (1) the participant’s overall performance and (2) the “average” performance of all participants. In the stress induction condition, the time limit, represented by a blue progress bar, is dynamically modulated to be 10% shorter than the participant’s average time required to complete previous trials. In the control condition, participants complete arithmetic problems of a comparable difficulty level without the time restriction or social evaluative performance displays. During the rest condition, the visual interface is displayed but no arithmetic problems are presented. The task is administered in three 5-min runs consisting of six 50-s blocks (2 per condition). After each run, participants are given scripted, negative feedback of their performance. After the task is complete, participants are debriefed and informed that the task was designed to increase their stress level.

Participants who participate in the neuroimaging session will complete subjective and biological measures of stress response immediately prior to and following the fMRI paradigm as described in exploratory aim 4. The Subjective Distress Units Scale (SUDS [44]) is a scale of 0 to 10 for measuring the subjective intensity of disturbance or distress currently experienced by an individual. The short-form Spielberger State-Trait Anxiety Inventory (STAI [45]) has 6 items assessing state anxiety. Salivary cortisol samples will be collected at 3 time points during the week 4 visit (at the beginning of the visit, immediately before the fMRI paradigm, and immediately after the fMRI paradigm).

#### fMRI data processing

FSL 5.0 (www.fmrib.ox.ac.uk/fsl) will be used for the neuroimaging analyses. Motion correction will be carried out using the Motion Correction Linear Image Registration Tool (McFLIRT, Version 5.0) with the estimated motion parameters entered as covariates in the general linear model. Brain Extraction Tool (BET) will be used for non-brain tissue/skull removal. The images will be smoothed using a FWHM Gaussian kernel (5 mm) and high-pass filtered (100 s cutoff) in the temporal domain with the FMRI Expert Analysis Tool (FEAT, Version 6.0). The EPI images will first be registered to the MBW, then to the MPRAGE using affine linear transformations, and into standard MNI space. Registration to standard space will be refined by FSL’s FNIRT nonlinear registration.

### Plans to promote participant retention and complete follow-up {18b}

In order to promote participant retention and honor the participants’ time during the study, participants are able to earn up to $385 dollars if they complete all study visits. Participants will receive $30 for participating in the in-person screening session and $30 for the physical exam. Participants will receive $30 for the randomization session and increasing amounts for each monthly follow-up visit as follows: $35 at week 4, $40 at week 8, and $45 at week 12. At the final safety visit (week 16), participants will be compensated $50 and will receive a $75 completion bonus if they complete all study visits. Participants can earn an addition $50 if they are eligible and complete the brain imaging session that takes place during week 4. Participants will have parking fees covered by the study and bus fare up to $3.50 (equivalent to two bus rides). Missing data will be minimized by attempting to follow up with all randomized participants even if they withdraw from allocated treatment and to collect information on reasons of loss to follow-up which can help determine whether it is related to the outcomes. Additionally, in-person visits can be adjusted to be conducted over the phone if the participant is no longer able to come to the study site on UCLA campus.

### Data management {19}

Source documents include but are not limited to original documents, data, and records such as hospital/medical records (including electronic health records), clinic charts, laboratory results, data recorded in automated instruments, and pharmacy records, etc. This study will use an electronic data capture (EDC) eCRF system (Qualtrics) and paper source documents. Data will be transcribed from source documentation directly into a statistical program such as SPSS and will be subsequently double checked by another member of the research staff. Only questionnaire data will be entered directly into eCRF (i.e., without prior written or electronic record of data). Paper copies of the eCRFs will be available in the event that the EDC is not accessible at the time the questionnaire is being completed. The transcribed data will be consistent with the source documents, or the discrepancies will be explained. All entries, corrections, and alterations will be made by the investigator or other authorized study personnel. Subjects will be identified on eCRFs and paper source documents by a unique subject number. The subject number will be used if it becomes necessary to identify data specific to a single subject. Regulatory bodies, such as the study sponsor, Food and Drug Administration (FDA), and Institutional Review Board (IRB), are eligible to review medical and research records related to this study as a part of their responsibility to protect human subjects in clinical research. Personal identifiers will be removed from photocopied or electronic medical and research records. To maintain subject confidentiality, research and clinical records will be stored in a locked cabinet. Only research staff, sponsor officials, and other required regulatory representatives will have access to the records. Subject information will not be released without written permission. The PI has received a Certificate of Confidentiality for this study.

In order to ensure compliance with protocol and regulatory guidelines, the PI will review study documents (including consent forms, data, and required reports) to verify their accuracy, completeness, and timeliness of collection and will provide feedback to staff on findings and on how to correct any errors found during the review. The PI will also designate appropriately qualified personnel to periodically perform quality assurance checks during and after the study. An independent Data and Safety Monitoring Board (DSMB) of external advisors will also meet prior to the start of the study, annually during enrolment and follow-up and at trial end to review safety data. See the “Frequency and plans for auditing trial conduct {23}” for more details.

### Confidentiality {27}

The risk of breach of confidentiality will be handled by emphasizing that information obtained during assessments and laboratory sessions is confidential and will be used solely for research purposes. All records will be kept in a locked file cabinet and will be available to research personnel who have been trained in human subjects’ protection guidelines. A cross-index of identified information will be kept in a separate locked location. In addition, all data will contain only a numeric code, all assessment procedures will be closely supervised by the faculty sponsor, and staff will be trained and reminded of the need to keep all information confidential. No names will be used in presenting data in lectures, seminars, and papers. Individual study participants will not be identified in any way in any presentation or publication of the study results and analysis of the results will be based on aggregate data only. Medical information will be released only with the expressed written consent of the subject. Lastly, the PI has obtained a Certificate of Confidentiality in order to further protect participant confidentiality during the study.

### Plans for collection, laboratory evaluation, and storage of biological specimens for genetic or molecular analysis in this trial/future use {33}

As outlined in exploratory aim 2, serum samples will be collected from all participants at randomization and at 4, 8, and 12-week follow-ups. Two lavender EDTA tubes will be collected for plasma to identify markers including innate immune receptors (TLR2, TLR4), cytokines (TNF-α, IL-1α, IL-1β, IL-2, IL-6, IL-10, IL12p70, GM-CSF, IFNγ), chemokines (MCP-1, MIP-1α, and MIP-1β), and other inflammatory signaling molecules (reactive oxidative species, NO, substance P, C-reactive protein). In addition, one RNA PAXgene tube will be collected at randomization and week 12 to identify transcription factors that code for cytokines.

As outlined in exploratory aim 4, salivary cortisol samples will be collected from participants who choose to participate in the neuroimaging session at 3 points during the week 4 neuroimaging visit: at the beginning of the visit and immediately before and after the fMRI session. Saliva will be collected via Salivette swab.

Biological samples will be stored in freezers (− 20). Serum samples will be assayed by a laboratory technician with extensive expertise processing biological samples for analyses of inflammatory markers. Salivary cortisol samples will be shipped to an outside lab for processing.

## Statistical methods

### Statistical methods for primary and secondary outcomes {20a}

#### Statistical analysis

Data analysis will utilize an intention-to-treat (ITT) population that includes all randomized patients who took at least one dose of medication and provided valid post-randomization outcome data. The primary tests of hypotheses will use percent heavy drinking days (4+ drinks for women/ 5+ drinks for men) measured by the TLFB at weeks 2, 4, 6, 8, 10, and 12 as a priori primary efficacy endpoint. Other outcomes will also be analyzed as described in the secondary and exploratory aims. Prior to statistical analyses, the data will be inspected to determine the advisability of scale transformations and to identify missing data, outliers, or other unusual features that may be influential. Preliminary analyses will also be performed to compare treatment groups on descriptive and clinical characteristics at baseline to ensure that randomization has succeeded. If confounding variables are found, they will be included as covariates in follow-up analyses.

### Comparisons

#### Primary aim 1

##### To test the primary hypothesis that IBUD (50 mg BID) will reduce percent heavy drinking days, as compared to placebo, over the course of the 12-week trial

The a priori primary efficacy endpoint will be percent heavy drinking days, defined as 4+ drinks for women/5+ drinks for men, measured bi-weekly during the maintenance phase of the study (weeks 1–12). Patients who discontinued medication will be allowed to remain in the study and participate in study assessments. The primary efficacy analysis will be performed using a repeated measures mixed effects model (GLIMMIX with PROC GLIMMIX in SAS) that includes treatment, time, treatment × time interaction, a random intercept and a random slope, and adjusts for other covariates such as demographic and baseline variables as appropriate. The mixed effects model approach permits testing of between-group differences, within-group changes, and performance trends over time. It also uses all observed repeated measurements data, treating the missing data mechanism as ignorable (see the discussion of attrition below). In addition to testing the treatment effects, a summary of least-square means, standard errors, and 95% confidence intervals (CIs) will be presented for each treatment and will be derived from fully adjusted models on untransformed outcomes averaged across the maintenance period.

#### Primary aim 2

##### To test the efficacy of IBUD (50 mg BID) on secondary alcohol consumption endpoints

In this aim, we plan on traditional analyses of the effects during the maintenance phase of the study (Weeks 1–12) on the following secondary alcohol consumption endpoints: (1) drinks per day, (2) drinks per drinking day, (3) percent days abstinent, (4) percent subjects with no heavy drinking days (PSNHDD), and (5) percent subjects abstinent. The analytical plan for the secondary outcomes with repeated measures are similar to that for the primary efficacy endpoint as discussed above for aim 1. For the dichotomous outcomes (PSNHDD and percent subject abstinent), logistic regression models will be used. Further, in light of recent research on AUD endpoints, we will examine secondary outcomes when allowing an optimal grace period of first 4 weeks and will evaluate the efficacy of IBUD over the maintenance period (weeks 5–12) [46].

#### Primary aim 3

##### To determine the effect of IBUD on neural activation to alcohol cues and psychosocial stress

Explanatory variables for the alcohol cues task will be created by convolving delta functions representing the onset of experimental events (alcohol, non-alcoholic beverage, blurred, and fixation cues; 24-s duration) with a double-gamma hemodynamic response function in FEAT. Temporal derivatives will be included as covariates. Second-level group analyses (medication condition) will then be conducted. The main contrast of interest will be activation during alcohol vs. non-alcoholic beverage blocks (ALC vs. BEV). Explanatory variables for the MIST will be created by convolving delta functions representing the onset of experimental conditions (stress, control, rest; 50-s duration) with a double-gamma hemodynamic response function in FEAT. Temporal derivatives will be included as covariates. Second-level analyses averaging over the three task runs will be conducted on the contrast images transformed into standard space. Third-level group analyses (medication condition) will then be conducted on the second-level images. The main contrast of interest will be activation during the stress vs. control blocks. For both tasks, *Z*-statistic images will be thresholded with cluster-based corrections for multiple comparisons based on the theory of Gaussian Random Fields with a cluster-forming threshold of *Z* > 2.3 and a cluster-probability threshold of *p* < 0.05.

#### Exploratory aim 1

##### To test whether the effects of IBUD (50 mg BID) on the primary and secondary endpoints (aims 1 and 2) are moderated by depressive symptomatology

We will examine if the effects of IBUD on the efficacy outcomes are moderated by depressive symptomatology. A moderator identifies for whom or under what conditions a treatment works. It may suggest which participants will respond most to treatment or identify subgroups with possibly different causal pathways. We will study moderators based upon criteria given in Kraemer et al. [47]. For the repeated measured efficacy outcomes, we will include depressive symptomatology in the analyses, using the mixed effects analysis designs described above, and testing the interactions of (depressive symptomatology) × treatment as well as (depressive symptomatology) × treatment × time. For the dichotomous outcomes (PSNHDD and percent subject abstinent), we will include depressive symptomatology in the logistic regression analyses and test the interactions of depressive symptomatology × treatment. To reduce confounding of main effects with these interaction terms and increase the interpretability of the regression coefficients, the variables will be centered as recommended by Kraemer and Blasey [48]. If interactions are significant, we will estimate treatment effects at low, middle, and high values of the moderator ([49], p. 175). Further, we will test whether physiological dependence (marked by tolerance and withdrawal) serves as a moderator of medication effects in this trial.

#### Exploratory aim 2

In this exploratory aim, we will test whether IBUD (50 mg BID) reduces neuroinflammation, as indexed by circulating blood levels of proinflammatory markers over the course of the 12-week trial, using the mixed effect model design. The statistical considerations are similar to exploratory aim 1 and thus not repeated here.

#### Exploratory aim 3

The percent signal change for BOLD activation in the ALC vs. BEV contrast detailed in primary aim 3 will be extracted from the left and right VS (anatomically defined). Percent heavy drinking days (PHDD), defined as 4+ drinks for women/5+ drinks for men, will be measured bi-weekly during the study. Two linear mixed models (left and right VS separately) with unstructured variance/covariance matrices (SPSS 22) will be used to evaluate the hypothesis that less VS activation to alcohol cues will predict fewer PHDD in IBUD-treated individuals. The model will include VS activation (ALC vs. BEV), a within-subject factor for time in the study, and a between-subject factor for medication. The dependent variable, PHDD, will be binned into 2-week periods following the scan (i.e., 6, 8, 10, and 12 weeks). Baseline covariates such as age, sex, and smoking status will be included in the model.

#### Exploratory aim 4

In this exploratory aim, we will test whether IBUD (50 mg BID) affects subjective (STAI; SUDS) and biological (salivary cortisol) measures of stress via three repeated measures over the course of one visit (at beginning of visit, immediately prior to fMRI session, and immediately following fMRI session). Statistical analysis will follow a similar mixed effect model design to what is used in exploratory aims 1 and 2; statistical considerations are thus also similar and not repeated here.

### Interim analyses {21b}

There are no plans to conduct interim analysis for this trial. However, safety data from this trial will be monitored on an ongoing basis. A summary report of all adverse events (AEs) for each of the first five subjects as they are completed will be prepared for review for the DSMB at least once every 6 months. If during the trial, a pattern of serious adverse events (SAEs) emerges, the PI will consult with the DSMB, the IRB, and with the NIAAA PO to determine whether the trial should continue as is or not.

### Methods for additional analyses (e.g., subgroup analyses) {20b}

As described in exploratory aim 1 above, we will study moderators in order to identify potential subgroups for whom IBUD may be a more or less effective treatment or with possibly different causal pathways. We will study moderators based upon criteria given in Kraemer et al. [47]. If interactions are significant, we will estimate treatment effects at low, middle, and high values of the moderator [49]. Further, we will test whether physiological dependence (marked by tolerance and withdrawal) serves as a moderator of medication effects in this trial.

### Methods in analysis to handle protocol non-adherence and any statistical methods to handle missing data {20c}

The intent-to-treat (ITT) principle requires all randomized participants to be included in the analyses. Missing data due to loss to follow-up will be dealt with through the following: First, we will minimize the extent of missing data by attempting to follow up all randomized participants event if they withdraw from allocated treatment and to collect information on reasons of loss to follow-up which can help to determine whether it is related to the outcome. Second, we plan to perform sensitivity analyses to explore the effect of departures from the missing data assumptions made in the efficacy analyses. Our primary efficacy model, the linear mixed effects model, assumes missing at random (MAR), which is plausible if the reason for missing data is administrative but implausible if missing data is outcome related. Sensitivity analysis choices will include, but are not limited to, imputation methods as well as the joint modeling approach. Imputation will be done in different ways including (a) imputing missing values as heavy drinking days and (b) multiple imputation similar to Litten et al. [21]. In addition, we will consider joint models that permit non-ignorable intermittent and monotone missing values [50].

### Plans to give access to the full protocol, participant-level data, and statistical code {31c}

After all data have been collected and the results of the study have been published, de-identified data will be made available to other qualified researchers on request, on a USB memory stick or other electronic means that is compatible with our systems and the investigator’s system. The request will be evaluated by the PI to ensure that it meets reasonable standards of scientific integrity and has the potential to make a reasonable scientific contribution. The UCLA-IRB-approved consent form asks participants to give permission to have their de-identified data shared with other scientists. Only data from participants who agree to data sharing will be used in the procedures outlined above.

## Oversight and monitoring

### Composition of the coordinating center and trial steering committee {5d}

Multiple review bodies provide oversight for the conduct of this research project. The most immediate of these review bodies is the local Human Subjects Committee, the UCLA Office of the Human Research Protection Program (OHRPP), and the Medical Institutional Review Board-3(MIRB-3), which oversees all psychiatry-related projects. The OHRPP, which is a division within the Office of Research Administration, provides the campus and the five UCLA Institutional Review Boards (IRBs) with professional guidance and administrative support, while the MIRB-3 reviews neuroscience, neurology, psychiatric, drug abuse, and related behavioral science research and dental research. The MIRB-3 has reviewed and approved the study protocol and will review and proposed study amendments. The MIRB-3 will also review the study on an annual basis, termed continuing review.

Other bodies charged with oversight for this study are:

1. NIAAA Program Officer—The NIAAA Program Officer will receive an annual summary of adverse events via progress note, as well as a phone call within 24 h of any unexpected, severe adverse events. If any pattern of severe adverse events emerges, the PI will consult with the NIAAA PO as well as the DSMB (below) and UCLA MIRB-3 to determine whether the trial should continue.
2. The UCLA Westwood Clinical and Translational Research Center (CTRC)—The medical screening visit clinical labs (Comprehensive Metabolic Panel and Complete Blood Count) to evaluate overall health and ECGs to screen for medical conditions that contraindicate taking ibudilast will be conducted at the outpatient unit of the UCLA CTRC. As well as, follow-up blood samples for neuroimmune assays will be completed at the CTRC or by a mobile CTRC nurse in our laboratory. Prior to initiation of studies using CTRC resources, protocol applications are reviewed. Following protocol approval, CTRC staff and PIs meet for a protocol discussion, after which study patients may be scheduled for CTRC visits.
3. The FDA Center for Drug Evaluation and Research, Division of Anesthetic, Critical Care and Addiction Drug Products—We have received an Investigational New Drug approval for the study of ibudilast (IND: #138,825), and we provide an annual report to the FDA with an update on study progress. In addition, an annual summary of AEs will be submitted to the FDA. We ensure that all studies meet or exceed FDA and ICH guidelines and regulations. All investigators in this study will promptly report all unexpected, severe adverse events to the PI, and the US FDA. A written report will be forwarded to the FDA within 10 working days of the incident and unexpected SAEs that occur with already-marketed medications.
4. The Data and Safety Monitoring Board (DSMB) assembled for this project (for additional details, please see the “Frequency and plans for auditing trial conduct {23}” section below).
  An independent DSMB of external advisors will meet prior to the start of the study, annually during enrollment and follow-up and at trial end to review safety data. The Board will be blinded to subjects’ actual randomized group assignments but may request at any time that the blind be broken by the data center, if concerns arise from the blinded data. In addition to annual meetings, the DSMB will meet after half of the subjects (66) have been randomized to review safety data and the integrity of the study (i.e., an evaluation of the dropout rate and impact on the planned statistical analysis of the data) and make a formal recommendation to the PI on the continuation or early stopping of the study due to safety concerns. Ad hoc meetings will be convened if SAEs occur that are considered at least possibly related to the study medication.

In the event of any major changes in the status of the ongoing protocol, the PI will inform the NIAAA program officer, DSMB, MIRB-3, CTRC, etc., immediately. Such changes would include:

1. Amendments to the protocol
2. Temporary suspension of patient accrual or of the protocol
3. Any change in informed consent or IRB approval status
4. Termination of patient accrual or of the protocol

### Composition of the data monitoring committee, its role, and reporting structure {21a}

An independent Data and Safety Monitoring Board (DSMB) will conduct regular audits over the course of the trial (see the “Frequency and plans for auditing trial conduct {23}” section for full details). We do not believe that an additional Data Monitoring Committee is needed for this study, as the trial presents minimal risk to participants.

### Adverse event reporting and harms {22}

An annual summary of adverse events (AEs) will be submitted to the FDA, the IRB, the DSMB, and NIAAA (via a progress report). The analysis of all adverse events accumulated-to-date will include a listing of all adverse events. Participants’ descriptions of adverse events from AE Forms will be grouped in some reasonable way, counted, and compared by treatment groups. A designation of “more common and drug-related” will be given to events occurring at an incidence of least 5% in subjects assigned to active drug, and for which the active drug incidence is at least twice the placebo incidence. Other significant (non-severe) adverse events that will be reported include the following: (1) marked abnormalities in laboratory, (2) vital signs, (3) electrocardiograms or other parameters, and (4) adverse dropouts and adverse events that lead to dose adjustments or to the addition of concomitant therapy.

The study physician will be available to participants for the entire duration of the study. Participants will have access to her 24-h pager and will report on adverse events at each monthly visit. The study physician will call every participant at the end of the first week on the study medication to discuss and manage any adverse events. Study staff will notify the study physician of any adverse events recorded during the follow-up visits. Side effects will be collected through (a) an open-ended question asking participants to report of any adverse events they may be experiencing and (b) a questionnaire-based assessment, the Systematic Assessment for Treatment Emergent Events (SAFTEE) [51, 52], which will be administered at the 4, 8, and 12-week follow-up visits. To continuously monitor safety, clinical labs will be repeated at each in-person follow-up visit (weeks 4, 8, and 12) and abnormal results will be discussed with the study physician.

### Frequency and plans for auditing trial conduct {23}

The PI will designate appropriately qualified personnel to periodically perform quality assurance checks at mutually convenient times during and after the study. These monitoring visits provide the opportunity to evaluate the progress of the study and obtain information about potential problems. The monitor will assure that data are accurate and in agreement with any paper source documentation used, verify that subjects’ consent for study participation has been properly obtained and documented, confirm that research subjects entered into the study meet inclusion and exclusion criteria, verify that study procedures are being conducted according to the protocol guidelines, monitor review AEs and SAEs, perform drug accountability, and assure that all essential documentation required by Good Clinical Practices (GCP) guidelines are appropriately filed. At the end of the study, they will confirm that the site has the appropriate essential documents on file, advise on storage of study records, and inspect the return and destruction records for unused study medication. An independent Data and Safety Monitoring Board (DSMB) of external advisors will meet prior to the start of the study, bi-annually during enrollment and follow-up and at trial end to review safety data. In addition to bi-annual meetings, the DSMB will meet after half the subjects (66) have been randomized to review safety data and the integrity of the study (i.e., an evaluation of the dropout rate and impact on the planned statistical analysis of the data) and make a formal recommendation to the PI on the continuation or early stopping of the study due to safety concerns.

The DSMB will provide periodic (bi-annual) review of the protocol, which is consistent with current practices at the UCLA CTRC, where this study will take place. The DSMB will provide comprehensive and regular input into whether there are appreciable changes to subjects’ risks to participation while the study is ongoing. The DSMB will monitor the following six aspects of study execution: (1) Administrative/Regulatory Updates, (2) Study Updates, (3) Quality Assurance and Safety Monitoring Procedures, (4) Study Accrual, (5) Protocol Violations/Deviations, and (6) Safety and Outcomes. After reviewing all these elements of the study, the DSMB will provide recommendations regarding safety/ethical concerns, study continuation, and protocol modifications. In addition, the DSMB will assist the PI and Study Physician to evaluate whether an active subject should be discontinued from further participation in the study for safety reasons.

### Plans for communicating important protocol amendments to relevant parties (e.g., trial participants, ethical committees) {25}

The PI will promptly inform the NIAAA Program Officer of any changes in recruitment or in the protocol that are relevant to safety, as well as any actions taken by the IRB as a result of its continuing review of the study. All necessary protocol changes will be submitted in writing as protocol amendments to the IRB by the PI for approval prior to implementation. In the event of any major changes in the status of any ongoing protocol, the PI will inform the NIAAA Program Officer, DSMB, IRB, CTRC, etc., immediately. Such changes would include amendments to the protocol, temporary suspension of patient accrual or of the protocol, any changes in informed consent or IRB approval status, and termination of patient accrual or of the protocol.

## Dissemination plans {31a}

Trial results will be communicated primarily via publication. Medicinova will review the manuscript prior to submission for publication but will have no influence on results or interpretation.

## Discussion

IBUD is a promising treatment for AUD as a neuroimmune modulator that has shown robust safety and early efficacy. In a preliminary study conducted by our lab (R21 AA022214; NCT02025998), IBUD was generally safe and well-tolerated, with no study dropouts or dose reductions over the course of the protocol, in a population with mild-to-severe AUD. The current study is supported by these early clinical results, as well as by compelling preclinical data validating its molecular targets and effects on alcohol phenotypes in animal models.

To the best of our knowledge, from a search of ClinicalTrials.gov as of May 16, 2020, at present the only registered trials of ibudilast for the treatment of alcohol use disorder have come from our lab, and the current study is the only interventional large-scale randomized clinical trial, testing IBUD in 132 treatment-seeking participants with AUD (NCT03594435). This single-center trial is double-blinded vs placebo, with 1:1 randomization.

Additionally, the current study goes beyond the scope of a standard clinical trial: in addition to assessing the effectiveness of the drug in AUD treatment, this study examines biological mechanisms underpinning the treatment, including neural activation to alcohol cues and stress, proinflammatory plasma biomarkers, and biological measures of stress response (i.e., salivary cortisol). Collection of these biological data for analyses of effects of IBUD represents an innovative aspect of the study which seeks to elucidate mechanisms underlying the treatment effects without compromising the clinical trial design required to address the primary aims. The ability of these data to provide mechanistic data for AUD pharmacotherapies is especially useful in the case of compounds like IBUD, for which the mechanism of action is currently unknown.

Some limitations are to be acknowledged. Standard face-to-face behavioral support (i.e., counseling) is not offered, instead using the computer-based Take Control program. While a recent study [19] comparing computer-delivered Take Control to Therapist-Delivered Platforms (TDP) found comparable drinking outcomes and higher medication adherence in the Take Control trials, suggesting that Take Control is a comparable and cost-efficient alternative to TDP in clinical trials, we acknowledge that face-to-face counseling remains the standard of care for AUD. Furthermore, abstinence is not the primary endpoint for the trial. However, based on our previous findings that IBUD improved phasic mood during stress- and alcohol-cue exposures as well as reductions in tonic levels of alcohol craving [18], we decided that PHDD was a more appropriate primary outcome.

The successful completion of the current study will further develop IBUD, a promising novel compound with strong preclinical and safety data for AUD. In the case of encouraging results—i.e., if IBUD proves superior to placebo in this study—the stage will be set for a confirmatory multi-site trial leading to FDA approval of a novel AUD treatment.

### Trial status

Recruitment began in October 2018. The first participant was randomized in October 2018, and as of the end of May 2020, 46 participants have been included. Recruitment is ongoing and aims to be completed by September 2021. The current protocol is version 8 created in September 2019 and approved by IRB on October 8, 2019. Any protocol modifications will be communicated to relevant parties (e.g., trial participants) and published on relevant channels (e.g., Clinicaltrials.gov). The results of this trial will be published in an appropriate scientific journal.

## Supplementary information

**Additional file 1: Table S1.** All items in the WHO trial registration data set {2b}.

**Additional file 2.**

## Data Availability

Prior to publication, only the investigative team will have access to the trial dataset. After all data have been collected and the results of the study have been published, de-identified data will be made available to other qualified researchers on request, on a USB memory stick or other electronic means that is compatible with our systems and the investigator’s system. The request will be evaluated by the PI to ensure that it meets reasonable standards of scientific integrity and has the potential to make a reasonable scientific contribution.

## Abbreviations

ACE: Adverse Childhood Experience Questionnaire
ADS: Alcohol Dependence Scale
AE: Adverse event
APT: Alcohol Purchase Task
AUD: Alcohol use disorder
AUDIT: Alcohol Use Disorder Identification Test
BAI: Beck Anxiety Inventory
BDI-II: Beck Depression Inventory-Revised
BOLD: Blood-oxygen-level-dependent
BrAC: Breath alcohol concentration
BTQ: Brief Trauma Questionnaire
CBC: Complete blood cell count
CI: Confidence interval
CIWA-R: Clinical Institute Withdrawal Assessment for Alcohol-Revised
CMP: Comprehensive metabolic panel
C-SSRS: Columbia Suicide Severity Rating Scale
CTMS: Clinical Trials Management System
CTRC: Clinical Translational Research Center
CUDIT: Cannabis Use Disorder Identification Test
DSMB: Data and Safety Monitoring Board
ECG: Electrocardiogram
EDC: Electronic data capture
FDA: Food and Drug Administration
fMRI: Functional magnetic resonance imaging
FTND: Fagerstrom Test for Nicotine Dependence
FTQ: Family Tree Questionnaire
GCP: Good Clinical Practice
GCPS: Graded Chronic Pain Scale
GGT: Gamma-glutamyl transferase
IBUD: Ibudilast
IRB: Institutional Review Board
ISI: Insomnia Severity Index
ITT: Intent-to-treat
LFT: Liver Function Test
MAR: Missing at random
MBW: Matched bandwidth
MIRB-3: Medical Institutional Review Board 3
MIST: Montreal Imaging Stress Task
MMIF: Macrophage Migration Inhibitory Factor
MPRAGE: Magnetization-Prepared Rapid-Acquisition Gradient Echo
OHRPP: Office of the Human Research Protection Program
PO: Program Officer
PACS: Penn Alcohol Craving Scale
PDE4/PDE10: Phosphodiesterases 4 and 10
PHDD: Percent heavy drinking days
POMS: Profile of Mood States
PSNHDD: Percent subject no heavy drinking days
RCT: Randomized clinical trial
RTC: Readiness to Change
SAE: Serious adverse event
SAFTEE: Systematic Assessment for Treatment Emergent Events
SCID-5: Structured Clinical Diagnostic Interview for DSM-5
STAI: Spielberger State Trait Anxiety Inventory
SUDS: Subjective Units of Distress Scale
TDP: Therapist-Delivered Platform
TLFB: Timeline follow-back
UCLA: University of California, Los Angeles
