# Supplementary material for: Ibudilast for alcohol use disorder: study protocol for a phase II randomized clinical trial
Source: Trials. 2020 Sep 11;21:779. doi: 10.1186/s13063-020-04670-y (PMC7488583; doi:10.1186/s13063-020-04670-y)
Supplement: Supplementary file 1 — Additional file 1: Table S1. All items in the WHO trial registration data set {2b}. [file 13063_2020_4670_MOESM1_ESM.docx]

**Supplementary Table 1. All items in the WHO trial registration data set {2b}.**

| Data Category | Information |
| --- | --- |
| Primary registry and trial identifying number | ClinicalTrials.gov  NCT03594435 |
| Date of registration in primary registry | July 20, 2018 |
| Secondary identifying numbers | none |
| Source(s) of monetary support | NIH NIAAA R01AA026190 |
| Primary sponsor | NIH NIAAA |
| Secondary sponsor(s) | Medicinova |
| Contact for public queries | Lara A. Ray, PhD. [lararay@psych.ucla.edu] |
| Contact for scientific queries | Lara A. Ray, PhD. University of California, Los Angeles, Psychology Department |
| Public title | Ibudilast for the Treatment of Alcohol Use Disorder |
| Scientific title | A Randomized Controlled Clinical Trial of Neuroimmune Modulator Ibudilast for the Treatment of Alcohol Use Disorder |
| Countries of recruitment | United States |
| Health condition(s) or problem(s) studied | Alcohol Use Disorder (AUD) |
| Intervention(s) | Active comparator: Ibudilast (IBUD), 50mg BID (5 x 10mg capsules twice daily) + Take Control behavioral support program.  Placebo comparator: matching capsules containing no active ingredient + TakeControl behavioral support program. |
| Key inclusion and exclusion criteria | Ages eligible for study: 18-65 years Sexes eligible for study: both Accepts healthy volunteers: no Inclusion criteria: meet current diagnostic criteria for AUD (moderate or severe); seeking treatment for AUD; report drinking at least 28 drinks per week if male, 21 per week if female, in the 28 days prior to consent. Exclusion criteria: current diagnosis of substance use disorder other than alcohol or nicotine; lifetime diagnosis of schizophrenia, bipolar disorder, or any psychotic disorder; positive urine screen for narcotics/amphetamines/sedative hypnotics; score ≥ 10 on withdrawal assessment; pregnancy, nursing, or refusal to use reliable birth control (if female); medical conditions that may interfere with safe study participation, at discretion of study physicians; prescription medication that contraindicates use of IBUD; current medications for AUD or psychotropic medications. Exclusion criteria for neuroimaging component: non-removable ferromagnetic object in body; claustrophobia; serious head injury. |
| Study type | Interventional Allocation: randomized Intervention model: parallel assignment Masking: double blind (subject, caregiver, investigator, outcomes assessor) Primary purpose: treatment Phase II |
| Date of first enrollment | October 2018 |
| Target sample size | 132; subset of 64 for neuroimaging component |
| Recruitment status | Recruiting |
| Primary outcome(s) | Primary study outcomes include: Percent heavy drinking days (≥5 drinks for men, ≥4 for women); drinks per day, drinks per drinking day, percent days abstinent, percent subjects with no heavy drinking days, percent subjects abstinent.  Primary neuroimaging outcomes include: BOLD activation differences during alcohol vs. non-alcoholic beverage blocks of cue-reactivity task and BOLD activation differences during stress vs. control blocks of psychosocial stress task. |
| Secondary outcome(s) | Alcohol craving and depressed mood; serum levels of immune receptors, cytokines, chemokines, and other inflammatory signaling molecules; association between percent signal change BOLD activation in alcohol vs. non-alcoholic beverage blocks of cue-reactivity task within ventral striatum region of interest and percent heavy drinking days; subjective and biological measures of stress response. |
