# Supplementary material for: Ibudilast for alcohol use disorder: study protocol for a phase II randomized clinical trial
Source: Trials. 2020 Sep 11;21:779. doi: 10.1186/s13063-020-04670-y (PMC7488583; doi:10.1186/s13063-020-04670-y)
Supplement: Supplementary file 2 — Additional file 2. [file 13063_2020_4670_MOESM2_ESM.pdf]

UNIVERSITY OF CALIFORNIA, LOS ANGELES
CONSENT TO PARTICIPATE IN RESEARCH

A RANDOMIZED CONTROLLED CLINICAL TRIAL OF THE NEUROIMMUNE MODULATOR IBUDILAST FOR THE TREATMENT OF ALCOHOL USE DISORDER
"A Study of the Experimental Medication Ibudilast for the Treatment of Alcohol Problems"

EXPERIMENTAL CONSENT

INTRODUCTION

You are asked to participate in a research study conducted by Lara Ray, PhD, from the Department of Psychology at the University of California, Los Angeles. The physicians in this study are Karen Miotto, MD, and Artha Gillis, MD, from the Department of Psychiatry and Biobehavioral Sciences at UCLA.

The researchers will explain this study to you. *Research studies are voluntary and include only people who choose to take part.* Please take your time about deciding whether to participate in this study. Before deciding:

- You can discuss this study with friends and family.
- You can also discuss it with your health care doctor or request a second opinion.
- If you have any questions, you can ask the researchers for more information before deciding to participate.

You have been asked to participate in this study because you are between the ages of 18 and 65, you have indicated the desire to reduce your drinking or get treatment for your alcohol use, and you passed the initial screening visit for the study. Your decision to participate or not to participate in this study will in no way affect your grades, employment status or standing with the University.

WHY IS THIS STUDY BEING DONE?

The purpose of this study is to see if the study medication, ibudilast, compared to placebo (a sugar pill), is useful for treating alcohol use disorder. The study consists of an in-person screening and physical exam, a 12 week treatment period including 4 in-person study visits, and one additional follow up visit one month after completing treatment. This study is being funded by the National Institute of Alcohol Abuse and Alcoholism (NIAAA).

REQUIREMENTS FOR TAKING PART IN TODAY'S STUDY VISIT

As was indicated in the prior visit's consent form, the study team will ask you to blow into a breathalyzer to make sure you are abstinent from alcohol and will ask you to provide a urine sample for a drug test (and pregnancy test, if female) before signing this consent form. You must meet the following requirements in order to take part in today's study visit:

- Produce a 0.000 on the breathalyzer. Please note, you may be asked to remain in the lab until the Investigator determines it is safe for you to leave if your breathalyzer result is above 0.000.
- Test negative on the urine drug screen for any drugs besides marijuana.
- Test negative on the pregnancy test.

If you do not meet these requirements we will NOT run your visit and you will NOT be compensated; however, you may have the opportunity to reschedule the visit at a later date.

WHAT WILL HAPPEN IF I TAKE PART IN THIS RESEARCH STUDY?

Physical Exam

If you sign the consent form, you will be asked to complete a physical exam with the study physician. The physician will interview you about your personal and family health history. Then, about 2 tablespoons of blood will be drawn from your arm by a medical professional at the UCLA Clinical and Translational Research Center (CTRC). Blood samples will be collected to evaluate your overall health, including tests of your blood sugar levels and kidney and liver function. An EKG will also be performed to rule out cardiovascular disease. The results from your physical, including your personal and family history and lab results, will remain confidential. If you pass the physical exam you will be invited to take part in the experimental sessions detailed below. If you do not pass the medical screening, you will not be allowed to participate in the study but will be offered referrals for alcohol treatment in the community.

Medication Procedures

If you are eligible and decide to participate in the study following the physical exam, you will be asked to attend an in-person visit to start the study medication (either ibudilast or placebo) and you will be on the medication for a total of 12 weeks. You will be asked to take 2 capsules (20 mg) twice a day for the first 2 days. Starting on Day 3, you will take 5 capsules (50 mg) twice a day until week 12. For the last 3 days of week 12, your dose will be reduced back down to 2 capsules (20 mg) twice a day prior to stopping the medication at the end of week 12. There is a 50% chance that you will be taking the active medication and a 50% chance that you will be taking placebo. You will not be told whether you are receiving placebo or ibudilast. At each visit, you will be provided enough medication to last until your next scheduled visit.

The medication being tested, ibudilast, is formulated as a 10 mg, extended-release, white capsule, and is taken by mouth. This medication works on the brain’s immune function and has been approved for over 25 years in Japan and Korea for the treatment of asthma and dizziness following a stroke. Ibudilast is not approved by the FDA for the treatment of alcohol use disorder. Placebo is an inactive substance or “sugar pill”, containing no medication. It is prepared as a white capsule, to look the same as the active medication, and is taken by mouth.

Study Visits

You will be asked to attend in-person study visits at weeks 1, 4, 8, 12 and 16. At the week 1, 4 and week 8 study visits, you will receive enough study medication to last until your next scheduled visit. At every in-person visit, you will be asked to provide a urine specimen for drug testing and pregnancy (if female), perform an alcohol breathalyzer test, and complete questionnaires and interviews about topics such as your alcohol use and mood. In addition, blood will be drawn to monitor safety and collect neuroinflammation data. You will be asked to bring all study medication and used packaging to each visit to assess for medication compliance. At weeks 2, 6, and 10, you will be contacted by phone to complete a brief interview about your recent alcohol use.

Brain Imaging Session

If you sign the consent form and are found to be eligible for an MRI, you will be asked to complete a brain imaging session. You will be asked to complete the brain imaging session as part of your week 4 in-person study visit.

You will be asked to abstain from drinking alcohol prior to coming into the lab for the brain imaging session, which will be verified through a breathalyzer. Only participants with a blood alcohol concentration of zero will be allowed to complete the scanning visit. Female participants will be given a pregnancy test to make sure that they are not pregnant. Pregnant women will not be allowed to participate in the study. We will also collect a urine sample on that day to verify compliance with the study medication. We will then ask you to fill out a few questionnaires including information about your use of alcohol.

After the initial questionnaires, you will receive some training on how to complete questionnaires in the scanner. The scanning will be performed at the Brain Mapping Center or at the Center for Cognitive Neuroscience, both located on the UCLA campus. You will be asked to lie down on a padded table, and your head will be placed in the center of a large, metal doughnut-shaped magnet. While the machine is running, you will hear loud banging noises. You will be offered earplugs to reduce the noise made by the magnet. Head and back support will also be provided to minimize discomfort. During part of the scan, you will not be asked to do anything other than remain still in the scanner for a few minutes. In the scanner, you will view images of alcohol and neutral cues and will be asked to rate your urge to drink alcohol. During the scan you will also be asked to solve a series of mental arithmetic problems and will be given feedback on your performance. You will be asked to provide 3 samples of saliva before and after the scan for testing of cortisol levels.

<u>Take Control Behavioral Platform</u>
The behavioral platform "Take Control" will consist of a series of 11 computerized modules, which you will view over 4 in-person study visits. If a visit is missed, missed modules will be reviewed at the next visit. The intervention is derived from a self-help approach developed by NIAAA that provides evidence-based, field tested information for individuals with alcohol problems, and suggestions for making changes in their drinking. The NIAAA material is publicly available in a NIAAA booklet entitled "Rethinking Drinking" and on the NIAAA website.

HOW LONG WILL I BE IN THE RESEARCH STUDY?

The physical exam will take about 1-1.5 hours. In-person study visits at weeks 4, 8 and 16 will last approximately 1-2 hours. The optional brain imaging session at week 4 will add an additional 1-1.5 hours to that visit. Each phone interview (at weeks 2, 6, and 10) will take approximately 10 minutes.

WHAT KIND OF RISKS OR DISCOMFORTS COULD I EXPECT?

The following complications or risks have been reported, or are known and may occur:

<u>Questionnaires:</u> There is some risk that you may find that certain questions make you uncomfortable. For example, you will answer a number of questions regarding your drinking and mood. You may refuse to answer any question.

<u>Loss of confidentiality</u>: As this study involves the use of your identifiable, personal information, there is a chance that a loss of confidentiality will occur. The researchers have procedures in place to lessen the possibility of this happening (see confidentiality section below).

<u>Ibudilast:</u> The most commonly observed adverse reactions are nausea followed by diarrhea and thirdly, vomiting. However, there appears to be some adaptation to these side effects within 1-2 weeks

of dosing. Less frequent adverse events include rash, itching sensation, dizziness, headache, tremors, insomnia, drowsiness, sudden blushing, weight loss, abdominal pain, indigestion, abdominal bloating, gastric ulcer, palpitations, hypotension, anemia, low white blood cell count, elevated liver function tests, lethargy, ringing in the ears, facial swelling, floating sensation and impaired taste. You will be asked to contact the study physician if you experience any discomfort because of the medication.

<u>Alcohol Withdrawal</u>: Some people may experience symptoms of alcohol withdrawal if they stop drinking suddenly. You will be informed about alcohol withdrawal during today's visit and monitored carefully throughout the study in case you begin to have symptoms of alcohol withdrawal. If you experience any of these symptoms, you will be instructed to call the study physician using the 24-hour phone number provided to you. The physician will ask you questions to determine if you are having serious withdrawal symptoms and will arrange for medical help if needed. During your in-person study visits throughout the study, you will be assessed for withdrawal symptoms you might be experiencing. You will be asked about changes in your health and drinking status. If you experience significant withdrawal symptoms, you will be asked to come to the study site or report to your local emergency room for further evaluation.

<u>Blood Draw:</u> The insertion of the needle poses a few minor risks. Minor bruising is possible due to the needle puncture (1-2% of people experience bruising). Also, lightheadedness, dizziness, rapid drop in blood pressure, sweating may occur during the insertion of the catheter (less than 1% of people experience this response). There is also the risk of fainting during the needle puncture and the risk of developing an infection at the IV site. The following measures will be taken to reduce possible risks and discomforts associated with the testing procedure and blood draw: (A) a medical screening session will ensure that you are in good physical health and will exclude participants for whom the IV procedure and the study medication may be risky; (B) insertion of the needle will be done by a health professional at the Clinical and Translational Research Center; (C) vital signs will be measured during needle insertion; and (D) you will be reclined in a comfortable chair and the experimenter will distract your attention away from the needle.

<u>Brain Imaging</u>: You may become anxious when you are in the head-holder during the MRI scan, which is a tight, bucket-like space. The machine makes very loud banging noises while taking pictures; you will be wearing earplugs and headphones so your hearing is protected. There are no harmful effects from the functional magnetic resonance imaging, but some people undergoing this procedure become anxious and afraid of closed spaces. If this happens to you, you can stop this procedure at any time. If you are pregnant or think you may be pregnant, you should not take part in this research. If you have any metal clips or plates in your body, or if you have a pacemaker, please tell the investigator about it before going near the MRI.

<u>If you are having suicidal thoughts:</u>
In the event that you tell the research staff that you are thinking about killing yourself or you answer "yes" to a question about having thoughts about suicide, the investigator will ask you more questions about your thoughts. Depending on how intense your thoughts are or how much you feel like hurting yourself, the research staff may provide you with referrals for treatment, work with you to contact your personal physician, trusted family member, or therapist to discuss your thoughts of harming yourself: or work with you on a plan that may include getting you to a hospital for safety.

<u>Unknown risks and discomforts</u>: The experimental treatments may have side effects that no one knows about yet. The researchers will let you know if they learn anything that might make you change your mind

about participating in the study.

This research study may involve risks that are currently unforeseeable.

ARE THERE ANY BENEFITS IF I PARTICIPATE?

You may or may not benefit from treatment with ibudilast or from the information provided in the Take Control Behavioral Platform during the study. Otherwise, there are no benefits to study participants.

WHAT ARE THE ANTICIPATED BENEFITS TO SOCIETY?

This study will advance medication development as a treatment for heavy alcohol use. To date, there are very few medications approved for the treatment of heavy alcohol use and their efficacy is modest. This project will also add knowledge about the relationship between brain measures of craving for alcohol in response to medications designed to reduce such cravings. Currently, little information is available about these issues. The scientific community and individuals with alcohol problems are likely to benefit from the knowledge that will be gained in this study.

WHAT OTHER CHOICES DO I HAVE IF I DON'T WANT TO PARTICIPATE?

The only alternative to participation in this study is to <u>not</u> participate.

WHAT WILL HAPPEN TO MY BLOOD SAMPLES?

All tissue and/or fluid samples are important to this research study. Your sample will be owned by the University of California or by a third party designated by the University (such as another university or a private company). If a commercial product is developed from this research project, the commercial product will be owned by the University of California or its designee. You will not profit financially from such a product. On the checklist at the end of the consent form, you will be asked to indicate if you would permit part of this sample to be shared with other researchers. If you agree to have your sample shared with other researchers and later decide to withdraw, we may not be able to retrieve your sample from other researchers. The researcher is not required to store your sample indefinitely. Because your sample is de-identified, no personal genetic or medical information can be provided to you. You are entitled, however, to any general information developed from this study that may be helpful to the medical care of you or your family members. It is your responsibility to contact the researcher if you want this information.

WILL INFORMATION ABOUT ME AND MY PARTICIPATION BE KEPT CONFIDENTIAL?

The researchers will do their best to make sure that your private information is kept confidential. Because this part of the study takes place at the CTRC, which is part of the UCLA Health System, medical research information (such as lab results) may appear in your electronic health record and you will be asked to sign a separate form describing this authorization. Information about you will be handled as confidentially as possible, but participating in research may involve a loss of privacy and the potential for a breach in confidentiality. Study data will be physically and electronically secured. As with any use of electronic means to store data, there is a risk of breach of data security.

<u>Use of personal information that can identify you:</u> Your data will be coded. Coded samples are those collected without identifying information. There will be some personal information collected during the

research, such as sex, age, ethnicity, or health, but this information is not enough to identify you.

How information about you will be stored: All data are identified only by numeric code and are stored in locked filing cabinets, such that any information that you provide us will remain confidential. After data has been collected and coded using identification numbers only, ALL tracking information (including name, phone number, email address, and date of birth) will be destroyed at the end of the study and there will never be any link between the data (ID numbers) and any contact or identifying information. When the results of the research are published or discussed in conferences, no information will be included that would reveal your identity.

People and agencies that will have access to your information: The research team, authorized UCLA personnel (such as employees of the UCLA Health System), and regulatory agencies such as the Research Advisory Panel of California, other State Regulatory Agencies, or the Food and Drug Administration (FDA), may have access to study data and records to monitor the study. Research records provided to authorized, non-UCLA personnel will not contain identifiable information about you. Publications and/or presentations that result from this study will not identify you by name.

It is possible that other investigators conducting other research might request that data collected for this study be shared for further research. For example, investigators associated with the government agency supporting this study might make this request. Even if you agree that your data may be shared with other investigators, your name or other personal identifying information would not be revealed. Though your privacy is very important to us and we will use many safety measures to protect your privacy, it is possible that there may be unforeseen privacy risks. For example, although we would not put any personal identifying information about you in a shared database, someone in the future might find some way to link your medical information or other information collected for this study back to you even in the absence of your name or other personal identifying information. Alternatively, there could be violations to the security of the separate computer systems used to store the codes linking your information to you.

Certificate of Confidentiality: To help us protect your privacy, we have obtained a Certificate of Confidentiality from the National Institutes of Health. With this certificate, the research cannot be forced to disclose information that may identify you, even by a court subpoena, if any federal, state, or local civil, criminal, administrative, legislative, or other proceedings. The researchers will use the Certificate to resist any demands for information that would identify you, except as explained below:

> The Certificate cannot be used to resist a demand for information from personnel of the United States Government that is used for auditing or evaluation of federally funded projects of for information that must be disclosed in order to meet the requirements of the federal Food and Drug Administration.
>
> You should understand that a Certificate of Confidentiality does not prevent you or a member of your family from voluntarily releasing information about yourself or your involvement in this research. If an insurer, employer, or other person obtains your written consent to receive research information, then the researchers may not use the Certificate to withhold that information.
>
> The Certificate of Confidentiality does not prevent the researchers from disclosing voluntarily, without your consent, information that would identify you as a participant in the research project under the following circumstances: (a) you are a danger to yourself or others, which may require immediate psychological attention; or (b) during the course of the research child or elder abuse becomes known and needs to be reported to the proper authority.

How long information from the study will be kept: All de-identified research records will be maintained for at least 3 years after the completion of this study.

ARE THERE ANY COSTS FOR TAKING PART IN THIS STUDY?

The study sponsor will supply and pay for the cost of supplying and administering the study drug and the related laboratory tests. Neither you nor your insurance will be billed for your participation in this research study.

WILL I BE PAID FOR PARTICIPATING?

You will be paid up to $305 for your participation in the experimental portion of this study, plus an additional $50 if you are eligible for and complete the brain imaging session, as follows:

Medical Screening Visit:	$30
Week 1 (Randomization) Visit:	$30
In-Person Visit Week 4:	$35
Brain Imaging Session:	$50
In-Person Visit Week 8:	$40
In-Person Visit Week 12:	$45
In-Person Visit Week 16:	$50
Completion Bonus:	$75

In addition, you will be provided with free parking validation or bus tokens for attendance to each visit.

WHAT ELSE DO I NEED TO KNOW ABOUT MY PARTICIPATION IN THE STUDY?

Participation and Withdrawal: Your participation in this research is VOLUNTARY. If you choose not to participate, that will not affect your relationship with UCLA or your right to health care or other services to which you are otherwise entitled. If you decide to participate, you are free to withdraw your consent and discontinue participation at any time without prejudice to your future care at UCLA.

New findings: During the course of the study, you will be informed of any significant new findings (either good or bad), such as changes in the risks or benefits resulting from participation in the research or new alternatives to participation that might cause you to change your mind about continuing in the study. If new information is provided to you, your consent to continue participating in this study will be re-obtained.

CAN THE RESEARCHERS REMOVE ME FROM THIS STUDY?

The researchers may end your participation in this study for a number of reasons, such as if your safety and welfare are at risk, if you do not follow instructions or if you miss scheduled visits. The researchers or the study sponsor might also decide to stop the study at any time. The investigator, Dr. Lara Ray, will make the decision and let you know if it is not possible for you to continue. If you must drop out because the investigator asks you to (rather than because you have decided on your own to withdraw), you will be compensated for the portion of the study that you have completed.

WHO CAN I CONTACT IF I HAVE QUESTIONS ABOUT THIS STUDY?

The research team: If you have any questions, comments or concerns about the research, you can talk to the one of the researchers. Please contact: Dr. Lara Ray, Principal Investigator, UCLA Department of Psychology, 1285 Franz Hall, Box 951563, Los Angeles, CA 90095-1563, Telephone: (310) 794-5383, Email: lararay@psych.ucla.edu. You can reach the study physician, Dr. Karen Miotto outside of normal business hours by calling (310) 206-8477 x12372 or Dr. Artha Gillis, by calling (424) 259-7039.

UCLA Office of the Human Research Protection Program (OHRPP): If you have questions about your rights while taking part in this study, or you have concerns or suggestions and you want to talk to someone other than the researchers about the study, please call the OHRPP at (310) 825-7122 or write to: UCLA Office of the Human Research Protection Program, 10889 Wilshire Blvd, Suite 830, Los Angeles, CA 90095-1406.

Public Information about this Study: ClinicalTrials.gov is a website that provides information about federally and privately supported clinical trials. A description of this clinical trial will be available on http://www.ClinicalTrials.gov, as required by U.S. Law. This website will not include information that can identify you. At most, the website will include a summary of the results. You can search this website at any time.

WHAT HAPPENS IF I BELIEVE I AM INJURED BECAUSE I TOOK PART IN THIS STUDY?

It is important that you promptly tell the researchers if you believe that you have been injured because of taking part in this study. You can tell the researcher in person or call him/her at the number(s) listed above. If you are injured as a result of being in this study, UCLA will provide necessary medical treatment. The costs of the treatment may be covered by the University of California or the study sponsor (NIAAA) or billed to you or your insurer just like other medical costs, depending on a number of factors. The University and the study sponsor do not normally provide any other form of compensation for injury. For more information about this, you may call the UCLA Office of the Human Research Protection Program at 310-825-5344 or send an email to mirb@research.ucla.edu.

WHAT ARE MY RIGHTS IF I TAKE PART IN THIS STUDY?

Taking part in this study is your choice. You can choose whether or not you want to participate. Whatever decision you make, there will be no penalty to you and you will not lose any of your regular benefits.

- You have a right to have all of your questions answered before deciding whether to take part.
- Your decision will not affect the medical care you receive from UCLA.
- If you decide to take part, you can leave the study at any time.
- If you decide to stop being in this study you should notify the research team right away. The researchers may ask you to complete some procedures in order to protect your safety.
  If you decide not to take part, you can still get medical care from UCLA.

HOW DO I INDICATE MY AGREEMENT TO PARTICIPATE?

If you agree to participate in this study you should sign and date below. You will be given a copy of this consent form and the Research Participant's Bill of Rights to keep. Additionally, you will be asked to sign a separate form authorizing access, use, creation, or disclosure of health information about you. By signing, you agree that you have read the information above, which is printed in English, and that this is a language that you read and understand.

SIGNATURE OF STUDY PARTICIPANT

____
Name of Participant

______________________________________ _____________
Signature of Participant Date

SHARING SAMPLES/DATA

Please check the appropriate box below and initial:

☐ ______ I agree to have my tissue/fluid sample and study questionnaire data shared with other researchers.

☐ ______ I do not want my tissue/fluid sample and study questionnaire data shared with other researchers.

SIGNATURE OF STUDY PHYSICIAN OBTAINING CONSENT

____
Name of Study Physician

______________________________________ _____________
Signature of Study Physician Date (must be same as above)

You will be given a copy of this information to keep for your records.
